# Utilizing reclassification to explore characteristics and prognosis of KDIGO_SCr_ AKI subgroups: a retrospective analysis of a multicenter prospective cohort study

**DOI:** 10.1080/0886022X.2021.1997761

**Published:** 2021-12-03

**Authors:** Gui-Ying Dong, Jun-Ping Qin, Youzhong An, Yan Kang, Xiangyou Yu, Mingyan Zhao, Xiaochun Ma, Yuhang Ai, Yuan Xu, Xiuming Xi, Chuanyun Qian, Dawei Wu, Renhua Sun, Shusheng Li, Zhenjie Hu, Xiangyuan Cao, Fachun Zhou, Li Jiang, Jiandong Lin, Erzhen Chen, Tiehe Qin, Zhenyang He, Jihong Zhu, Bin Du

**Affiliations:** a Medical Intensive Care Unit, Peking Union Medical College Hospital, Beijing, China;; b Trauma Intensive Care Unit, Peking University People’s Hospital, Key Laboratory of Trauma and Neural Regeneration (Peking University); Ministry of Education, Beijing, China; c Department of Critical Care Medicine, Tsinghua changgung Hospital, Beijing, China; d Department of Critical Care Medicine, Peking University People’s Hospital, Beijing, China; e Department of Critical Care Medicine, West China Hospital, Sichuan University, Chengdu, China; f Department of Critical Care Medicine, First Affiliated Hospital, Xinjiang Medical University, Urumqi, China; g Department of Critical Care Medicine, The First Affiliated Hospital, Harbin Medical University, Harbin, China; h Department of Critical Care Medicine, The First Affiliated Hospital of China Medical University, Shenyang, China; i Department of Critical Care Medicine, Xiangya Hospital, Central South University, Changsha, China; j Department of Critical Care Medicine, Beijing Tongren Hospital, Capital Medical University, Beijing, China; k Department of Critical Care Medicine, Fuxing Hospital, Capital Medical University, Beijing, China; l Department of Emergency Medicine, The First Affiliated Hospital of Kunming Medical College, Kunming, China; m Department of Critical Care Medicine, Qilu Hospital, Shandong University, Jinan, China; n Department of Critical Care Medicine, Zhejiang Provincial People’s Hospital, Hangzhou, China; o Department of Critical Care Medicine, Tongji Hospital, Tongji Medical College, Huazhong University of Science & Technology, Wuhan, China; p Department of Critical Care Medicine, Hebei Medical University Fourth Hospital, Shijiazhuang, China; q Department of Critical Care Medicine, Affiliated Hospital of Ningxia Medical University, Yinchuan, China; r Department of Critical Care Medicine, The First Affiliated Hospital, Chongqing Medical University, Chongqing, China; s Department of Critical Care Medicine, Xuanwu Hospital, Capital Medical University, Beijing, China; t Department of Critical Care Medicine, The First Affiliated Hospital of Fujian Medical University, Fuzhou, China; u Department of Emergency Medicine, Ruijin Hospital, Shanghai Jiao Tong University, Shanghai, China; v Department of Critical Care Medicine, Guangdong General Hospital, Guangzhou, China; w Department of Critical Care Medicine, Hainan Provincial People’s Hospital, Haikou, China; x Department of Emergency Medicine, Peking University People’s Hospital, Beijing, China

**Keywords:** Acute kidney injury, KDIGO, intensive care unit, mortality, length of stay

## Abstract

**Background:**

Acute kidney injury (AKI) is widespread in the intensive care unit (ICU) and affects patient prognosis. According to Kidney Disease: Improving Global Outcomes (KDIGO) guidelines, the absolute and relative increases of serum creatinine (Scr) are classified into the same stage. Whether the prognosis of the two types of patients is similar in the ICU remains unclear.

**Methods:**

According to the absolute and relative increase of Scr, AKI stage 1 and stage 3 patients were divided into stage 1a and 1b, stage 3a and 3b groups, respectively. Their demographics, laboratory results, clinical characteristics, and outcomes were analyzed retrospectively.

**Results:**

Of the 345 eligible cases, we analyzed stage 1 because stage 3a group had only one patient. Using 53 or 61.88 µmol/L as the reference Scr (Scr_ref_), no significant differences were observed in ICU mortality (*P_53_*=0.076, *P_61.88_*=0.070) or renal replacement therapy (RRT) ratio, (*P_53_*=0.356, *P_61.88_*=0.471) between stage 1a and 1b, but stage 1b had longer ICU length of stay (LOS) than stage 1a (*P_53_*<0.001, *P_61.88_*=0.032). In the Kaplan-Meier survival analysis, no differences were observed in ICU mortality between stage 1a and 1b (*P_53_*=0.378, *P_61.88_*=0.255). In a multivariate analysis, respiratory failure [HR = 4.462 (95% CI 1.144–17.401), *p* = 0.031] and vasoactive drug therapy [HR = 4.023 (95% CI 1.584–10.216), *p* = 0.003] were found to be independently associated with increased risk of death.

**Conclusion:**

ICU LOS benefit was more prominent in KDIGO_SCr_ AKI stage 1a patients than in stage 1 b. Further prospective studies with a larger sample size are necessary to confirm the effectiveness of reclassification.

## Introduction

Acute kidney injury (AKI) comprises a heterogeneous group of conditions characterized by a sudden decrease in the glomerular filtration rate, manifested by an increase in serum creatinine concentration or oliguria, and classified by stage and cause [1]. AKI itself might independently increase mortality, and it is associated with other negative consequences, such as progression to chronic kidney disease, which may require renal replacement therapy (RRT), prolonged hospitalization, increased medical costs, and subsequent lower quality of life [2–7]. Studies have reported that in the intensive care unit (ICU) setting, AKI-associated morbidity and mortality rates are 55.38–57.3% and 25.8–26.9%, respectively [8–9]. Because serum creatinine (Scr) level is highly associated with the outcome in patients with AKI [10], international consensus criteria have been developed and later refined for the diagnosis and staging of AKI, the severity of which is classified according to urine output and elevations in Scr level [11–13]. The recent Kidney Disease: Improving Global Outcomes (KDIGO) clinical practice guidelines defined AKI stage 1 as the following: increase in Scr by ≥26.5 µmol/l within 48 h; or an increase in Scr to ≥1.5 times of baseline, which is known or presumed to have occurred within the preceding 7 days [11]. However, whether the prognosis of the two types of patients in AKI stage 1 is consistent in ICU remains unclear.

Recently, Sparrow et al. [14] evaluated the potential impact of further categorizing AKI stage 1 into two stages based on Scr criteria in a cohort of 81,651 inpatients, that is, AKI stage 1a as an absolute increase in Scr of 26.5 µmol/L (0.3 mg/dl) within 48 h and stage 1b as a 50% relative increase in Scr within 7 days. The authors found that patients with AKI stages 1a and 1b experienced clinically meaningful and statistically significant differences in length of stay (LOS) and mortality. This study suggests that a modified 2-stage version of the KDIGO AKI stage 1 may provide additional prognostic information.

At present, there is no detailed research on characteristics of hospitalized patients in China based on further categorizing AKI stages, especially ICU patients. Therefore, we aimed to investigate the influence of such a strategy of further categorizing AKI stage 1 on the clinical prognosis of AKI patients in the ICU setting based on the Chinese critical care trial group database.

## Methods

### Study design and setting

The prospective observational study was performed from 1 July 2009 to 31 August 2009 in 22 tertiary hospitals from 19 provinces and autonomous regions of China [15]. We conducted a retrospective study with information from this prospective cohort database. All patients who were admitted to the ICU followed the guidelines for the construction and management of critical care departments (Trial) issued by the Ministry of health of China in 2009 [16]. Initial database research was approved by the ethics committee of the Fuxing Hospital affiliated to Capital Medical University. The data, including patient records and information, were anonymized and de-identified prior to analysis. Written informed consent was waived because of the study design. The STROBE (Strengthening the Reporting of Observational Studies in Epidemiology) guideline recommendations were used as a reference [17].

### Standards and definitions

Baseline Scr (Scr_baseline_) refers to the Scr measured on hospital admission, while Scr_ref_ refers to the lowest Scr value within 48 h or 7 days, and peak Scr (Scr_peak_) refers to the highest Scr value when AKI is diagnosed.

**Figure 1.**
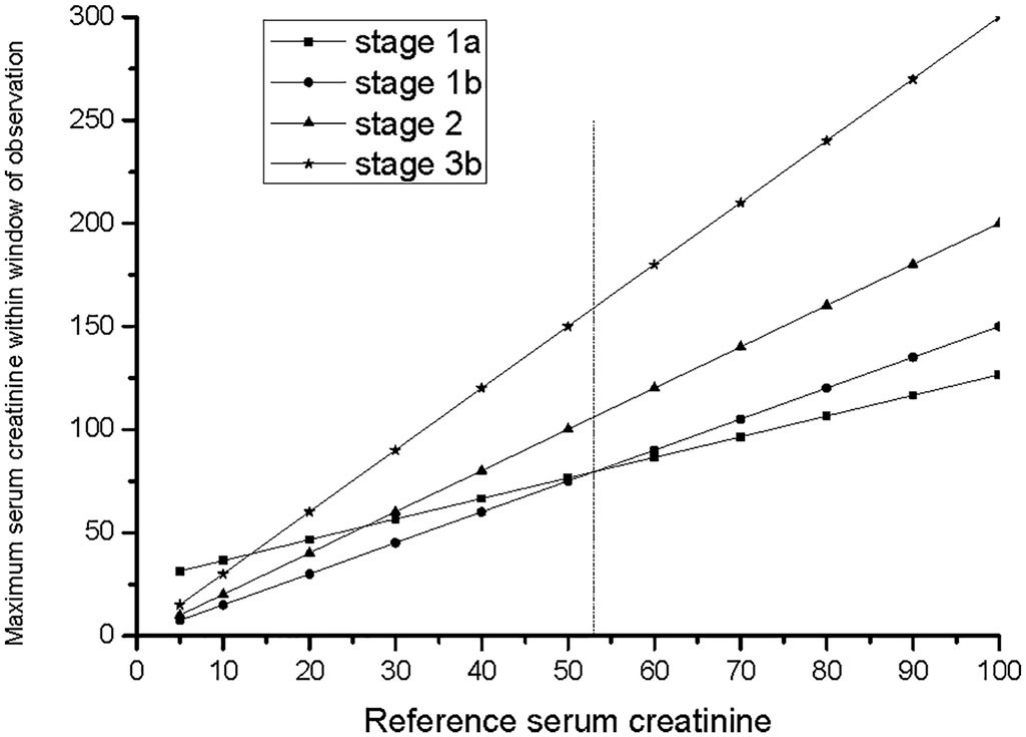
KDIGO_Scr_-AKI staging according to the formula. Calculations for Scr_ref_. When stage 1a: *x* + 26.5; stage 1b: 1.5x, if *x* + 26.5 = 1.5x, *x* = 53. When Scr_ref_ > 53 µmol/L, Scr_peak_ (1b) > Scr_peak_ (1a).

The proposed modifications to KDIGO AKI Scr (KDIGO_Scr_ -AKI) stage are shown in Table 1. According to the absolute or relative increase of Scr, stage 1 was divided into two subgroups: stage 1a and stage 1b. Stage 3 patients also followed this method and were divided into two subgroups: stage 3a and stage 3b. The Scr absolute increase of 26.5 µmol/L is equal to a 50% increase when Scr_ref_ is 53 µmol/L. According to the KDIGO_Scr_-AKI staging standard [11], stages can be rewritten as the formula: *y* = *kx* + *e*, stage 1a: *y* ≥ *x* + 26.5 and *y* < 1.5*x*; stage 1 b: *y* = *ax*, 1.5≤*a* < 2; stage 2: *y* = *bx*, 2 ≤*b* < 3; stage 3a: *y* = *xe* + 44.2, *x* ≥ 353.6; stage 3 b: *y* = *cx*, *c* ≥ 3. If *x* + 26.5 = 1.5*x*, then *x* = 53, so when Scr_ref_ >53 µmol/L,Scr_peak_ (1 b) > Scr_peak_ (1a), clinical features and outcomes of the two subgroups were compared (Figure 1). When Scr_ref_ >353.6 µmol/L, stage 3a and stage 3 b were compared. If a patient met stage 1a criteria on hospital day 2 and progressed to stage 1 b within 7 days, the patient was classified as stage 1 b.

**Table 1.** Staging of acute kidney injury according to KDIGO.

Stage	Serum creatinine (Scr)	Urine volume
1	(1) 1a,Scr increase o*f* ≥ 26.5umol/L (0.3 mg/dl) ; (2) 1b,Sc*r* ≥ 1.5–1.9 times baseline within 7 days	<0.5 ml*kg^−1^*h^−1^ for 6–12 h
2	Sc*r* ≥ 2.0 times baseline within 7 days	<0.5 ml*kg^−1^*h^−1^ fo*r* ≥ 12 h
3	(1) 3a,Sc*r* ≥ 353.6 µmol/l (4 mg/dl) and acute ris*e* ≥ 44.2 µmol/l(0.5 mg/dl); (2) 3b,Sc*r* ≥ 3.0 times baseline within 7 days; (3) Ag*e* < 18 years old, eGF*R* < 35ml*min^−1^*1.73 m^−2^; (4) Acute dialysis	<0.3 ml*kg^−1^*h^−1^fo*r* ≥ 24 h anuria fo*r* ≥ 12 h

In order to further illustrate whether the standard of our hypothesis is correct or not, we selected Scr_ref_ = 61.88 µmol/L [14] to reanalyze our data.

### Clinical variables

Demographic and clinical data were collected at ICU presentation, including gender, age, weight, acute physiology and chronic health evaluation II (APACHE II) score, sequential organ failure assessment (SOFA) score, Charlson comorbidity index (CCI) [18], estimated glomerular filtration rate (eGFR) [19], admission status, reasons for ICU admission, interventions during ICU, ICU LOS and comorbidities. Scr levels were recorded for one week after admission.

### Statistical analysis

SPSS 25.0 (SPSS Inc., Chicago, IL) and OriginPro8 mapping software (OriginLab Inc. Northampton, MA) were used for data analysis. Kolmogorov-Smirnov test was used to test for normality. Continuous variables were described as the median (interquartile range, IQR) and were compared by the Mann-Whitney *U* test. Categorical variables were presented as *n* (%) and were compared by the chi-square test or Fisher’s exact test. We assessed the risk of patients’ death based on refined stage 1 and using the Kaplan-Meier curves and the log-rank test, followed by multivariable-adjusted Cox proportional hazards models adjusted for covariates and potential confounders. All statistical analyses were performed using the bicaudal test, *p <* 0.05 was considered statistically significant.

## Results

### General information

**Figure 2.**
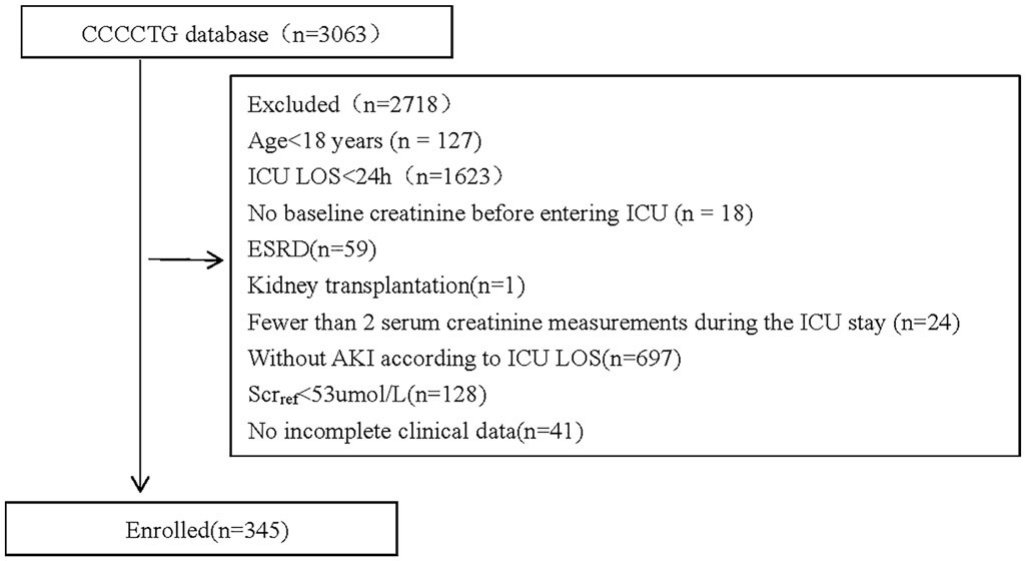
Patient flow chart illustrating enrollment of the study population. ICU: intensive care unit; LOS: length of stay; ESRD: end-stage kidney disease; CCCCTG, Chinese critical care clinical trial group. Scr_ref,_ the lowest Scr value within 48 h or 7 days when AKI is diagnosed. Patients with a Scr_ref_ < 53 µmol/L were excluded from this study because proposed AKI stages of 1a and 1b do not apply.

The flow diagram of the study is shown in Figure 2. From a total of 3063 records from database, reasons for exclusion included age ≤18 years (*n* = 127), ICU LOS <24 h (*n* = 1623), no Scr_baseline_ (*n* = 18), end-stage kidney disease (*n* = 59), kidney transplantation (*n* = 1), fewer than 2 Scr measurements in ICU (*n* = 24), without progression to AKI (*n* = 697), Scr_ref_ <53 µmol/L (*n* = 128) and incomplete clinical data (*n* = 41). Based on the existing database, the available sample size is 345 who were all enrolled in the final analysis.

The mortality of ICU-AKI was 13.91% (48/345). The distribution of AKI by stage was 145 (42.32%) stage 1, 72 (20.87%) stage 2, 128 (36.81%) stage 3. Within AKI subgroups, 44 (13.04%) were in stage 1a, 101 (29.28%) were in stage 1b, 1 (0.29%) was in stage 3a and 126 (36.52%) were in stage 3b. For statistical analysis, AKI was classified into stage 1a, stage 1b, stage 2 and stage 3.

### Demographics and characteristics of patients

The demographics and general characteristics of the 345 patients included in the study are reported in Table 2. The median age of the patients was 59 years (IQR 42–73), and 78.13% of the participants were male. The stage 1a patients had a median age 59 years (IQR 40–74) and 28 were men (63.64%). Stage 1b patients had a median age of 68 years (IQR 42–73) and 63 were men (62.38%). The average weight was 65 kg (IQR 59–70) in stage 1a patients and 65 kg (IQR 60–70) in stage 1b patients. Baseline data (demography, illness severity scores, comorbidities, admission status, reasons for ICU admission, and interventions in ICU, showed no significant differences between stage 1a and stage 1 b patients. The eGFR was significantly lower (median 98 mL/min/1.73 m^2^ [IQR 78–115] vs. 115 mL/min/1.73 m^2^ [IQR 91–141]; *p* = 0.009) and Scr_ref_ was significantly higher (median 84 µmol/L [IQR 69–98] vs. 73 µmol/L [IQR 61–89]; *p* = 0.020) in stage 1a patients than in stage 1 b patients.

**Table 2.** Clinical features of AKI patients (*n* = 345).

	All (*n* = 345)	Stage1a (*n* = 44)	Stage1b (*n* = 101)	Stage2 (*n* = 72)	Stage3 (*n* = 128)	*p* Value
Demographics						
Male	210 (78.13%)	28 (63.64%)	63 (62.38%)	40 (55.56%)	79 (61.72%)	0.885
Age (years)	59 (42, 73)	59 (40, 74)	68 (42, 73)	61 (42, 73)	58 (41, 73)	0.788
Weight (kg)	65 (56, 70)	65 (59, 70)	65 (60, 70)	65 (55, 70)	65 (55, 70)	0.962
Illness severity score						
APACHE II	16 (11, 23)	16 (10, 24)	17 (10, 23)	15 (10, 23)	16 (12, 23)	0.776
SOFA	6 (3, 9)	5 (2, 9)	5 (3, 9)	6 (4, 9)	6 (3, 8)	0.875
Comorbidity						
Chronic kidney disease	9 (2.61%)	1 (2.27%)	3 (2.97%)	2 (2.78%)	3 (2.34%)	1.000
Hypertension	91 (26.38%)	13 (29.55%)	29 (28.71%)	19 (26.39%)	30 (23.44%)	0.919
Diabetes mellitus	50 (14.49%)	9 (20.45%)	12 (11.88%)	12 (16.67%)	17 (13.28%)	0.177
Coronary artery disease	60 (17.39%)	8 (8.18%)	18 (17.82%)	11 (15.28%)	23 (17.97%)	0.959
Malignant tumor	42 (78.13%)	5 (11.36%)	13 (12.87%)	12 (16.67%)	12 (9.38%)	0.800
Chronic pulmonary disease	36 (12.17%)	5 (11.36%)	7 (6.93%)	11 (15.28%)	13 (10.16%)	0.373
Connective tissue disease	10 (2.9%)	1 (2.27%)	3 (2.97%)	2 (2.78%)	4 (3.13%)	1.000
CCI	1 (0, 2)	1 (0, 2)	0 (0, 2)	1 (0, 2)	0 (0, 2)	0.346
Admission status						
Emergency room	89 (25.80%)	11 (25.00%)	19 (18.81%)	23 (31.94%)	36 (28.13%)	0.398
General ward	115 (33.33%)	12 (27.27%)	37 (36.63%)	19 (26.39%)	47 (36.72%)	0.273
Postoperation	129 (37.39%)	19 (43.18%)	41 (40.59%)	26 (36.11%)	43 (33.59%)	0.771
Other ICU	12 (3.48%)	2 (4.55%)	4 (3.96%)	4 (5.56%)	2 (1.56%)	1.000
Reason for ICU admission						
Surgery	105 (30.43%)	16 (16.36%)	31 (30.69%)	18 (25.00%)	40 (31.25%)	0.502
Trauma	50 (14.49%)	6 (13.63%)	14 (13.86%)	12 (16.67%)	18 (14.06%)	0.768
Respiratory failure	55 (15.94%)	5 (11.36%)	18 (17.82%)	13 (18.06%)	19 (14.84%)	0.328
Heart failure	19 (5.51%)	1 (2.27%)	4 (3.96%)	8 (11.11%)	6 (4.69%)	0.986
Neurological system	35 (10.14%)	8 (18.18%)	12 (11.88%)	7 (9.72%)	8 (6.25%)	0.312
Sepsis	56 (16.23%)	4 (9.09%)	15 (14.85%)	12 (16.67%)	25 (19.53%)	0.498
Other	25 (7.25%)	4 (9.09%)	7 (6.93%)	2 (2.78%)	12 (9.38%)	0.912
Renal function						
eGFR (ml*min^-1^*1.73m^2^ )	110 (91, 134)	98 (78, 115)	115 (91, 141)	107 (92, 128)	118 (91, 138)	0.009*
Scr_ref_ (µmol/L)	74 (63, 90)	84 (69, 98)	73 (61, 89)	73 (66, 87)	72 (61, 88)	0.020*
Intervention during ICU stay						
Mechanical ventilation	247 (71.59%)	31 (70.45%)	70 (69.3%)	54 (75.00%)	92 (71.88%)	1.000
Vasoactive drug therapy	110 (31.88%)	15 (34.09%)	30 (29.7%)	23 (31.94%)	42 (32.81%)	0.600

AKI: acute kidney injury; APACHE II: acute physiology and chronic health evaluation II; SOFA: sequential organ failure assessment; CCI: the Charlson comorbidity index.

Data are presented as the median (IQR) or *n* (%). *p* was the comparison between stage 1a and stage 1b.

### Clinical outcomes in different subgroups of stage 1

**Figure 3.**
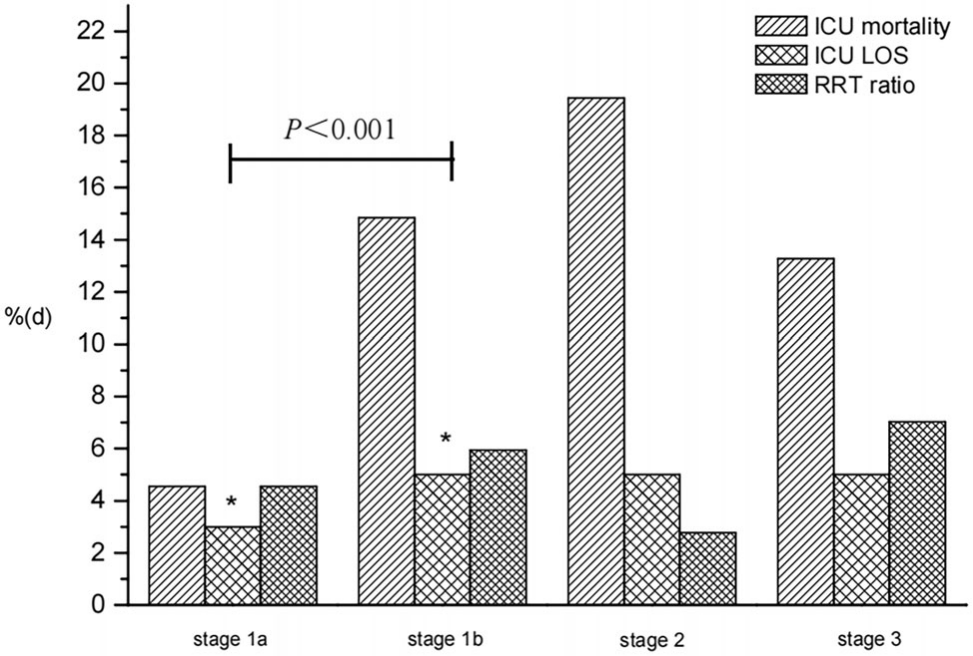
Clinical outcomes in different subgroups of stage 1 (Scr_ref_ = 53 µmol/L). ICU: intensive care unit; LOS: length of stay; RRT: renal replacement therapy.

Figure 3 shows that stage 1a patients had lower ICU mortality (4.55%) than stage 1b patients (14.85%; *p* = 0.076). Stage 1b patients required RRT support more often than stage 1a patients (5.94% vs. 4.55%, *p* = 0.356), but there was no significant difference in ICU mortality and RRT ratio between these two subgroups. The ICU LOS was lower in stage 1a patients than stage 1b patients (median 3 days [IQR 2–7] vs. 5 days [IQR 3–11]; *p* < 0.001).

### External validation

**Figure 4.**
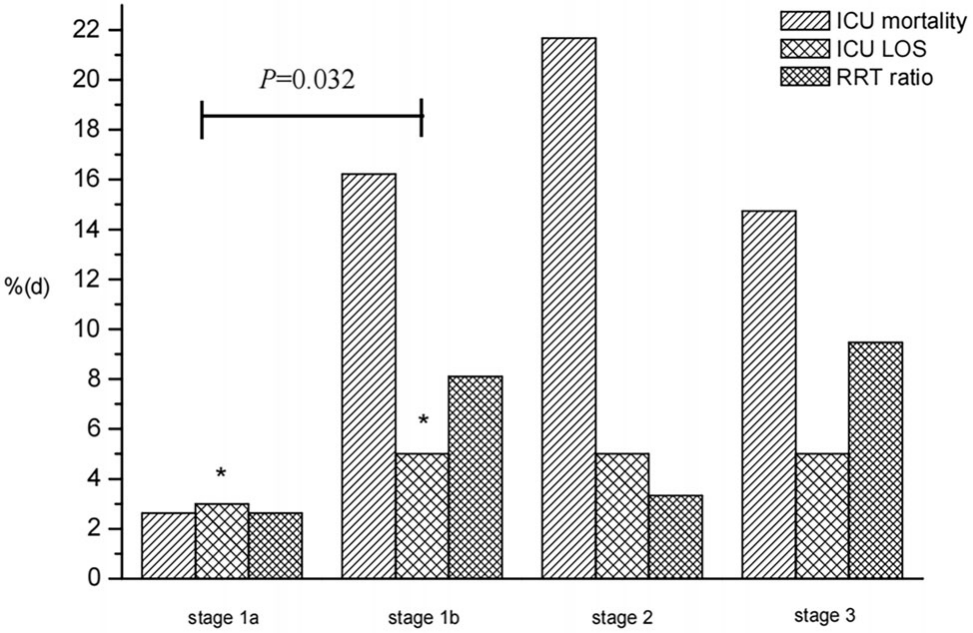
Clinical outcomes in different subgroups of stage 1 (Scr_ref_ = 61.88 µmol/L). ICU: intensive care unit; LOS: length of stay; RRT: renal replacement therapy.

Using Scr_ref_ = 61.88 µmol/L as the exclusion cutoff, stage 1b patients showed a longer median ICU LOS than stage 1a patients (median 5 days [IQR 3–11] vs. 3 days [IQR 2–7]; *p* = 0.032). There were no significant differences in ICU mortality (2.63% vs. 16.22%, *p* = 0.070) and RRT ratio (2.63% vs. 11%, *p* = 0.471) between the two subgroups (Figure 4).

### Survival analyses

**Figure 5.**
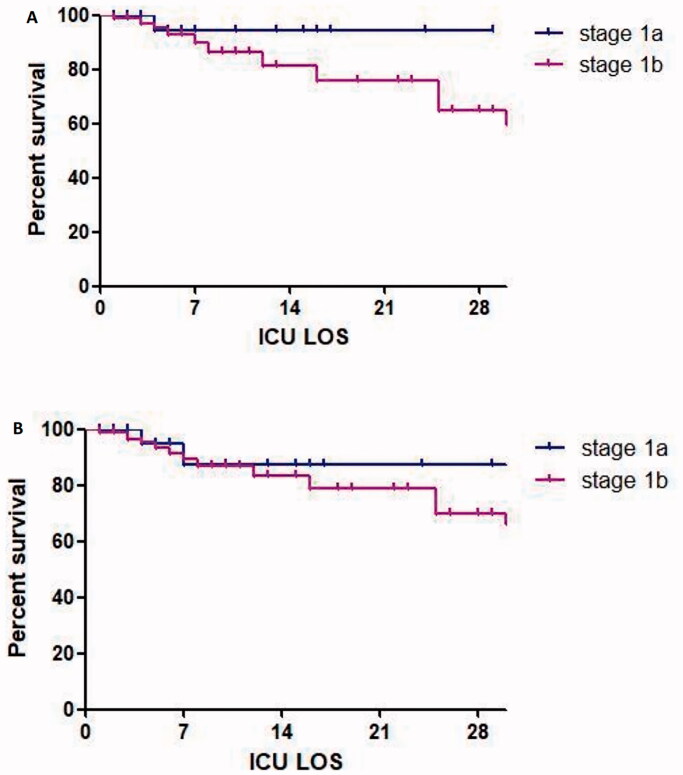
Univariate Kaplan–Meier curves for AKI stage 1 survival. A and B show comparisons of survival between stage 1a and stage 1b with log-rank *P_53_* 0.378 and *P_61.88_* 0.255, respectively.

Figure 5 demonstrates using the univariate Kaplan–Meier survival analysis, patient survival was not found to be statistically different between stage 1a and stage 1b patients (log-rank Test: *X*^2^ = 0.585, *p* = 0.378). This result remained non-significant after re-classification with Scr_ref_ = 61.88 µmol/L (log-rank Test: *X*^2^=4.056, *p* = 0.255).

We chose the multivariate Cox regression hazard model to test for differences in the hazard of death over 28 days according to refined stage 1, in order to allow for the correction of potential confounding factors including age, eGFR, APACHE II, SOFA, chronic kidney disease, CCI, sepsis, trauma, surgery and mechanical ventilation, respiratory failure and vasoactive drug therapy as covariates, which were found to be independently associated with ICU mortality from univariate Kaplan–Meier survival analysis. Respiratory failure and vasoactive drug therapy were found to be a significant independent predictors for ICU mortality during the study period [hazard ratio [HR] = 4.458 (95% confidence interval [CI] 1.141–17.413), *p* = 0.032; HR = 5.181 (95% CI 2.033–13.199), *p* = 0.001 respectively]. Other variables included in the analysis were not found to be independently associated with ICU mortality (Table 3).

**Table 3.** Multivariate Cox regression hazard model for ICU mortality.

	HR	95% CI	*p* Value
Vasoactive drug therapy	5.181	2.033–13.199	0.001*
Mechanical ventilation	1.67	0.408–6.833	0.476
Sepsis	1.371	0.154–12.213	0.778
Respiratory failure	4.458	1.141–17.413	0.032*
Trauma	1.213	0.309–4.755	0.782
Surgery	1.519	0.435–5.304	0.512
CCI	0.849	0.6–1.203	0.358
Chronic kidney disease	1.097	0.227–5.291	0.909
SOFA	0.955	0.853–1.07	0.43
APACHE II	1.034	0.984–1.087	0.189
eGFR	1.002	0.99–1.014	0.795
Age	0.972	0.943–1.002	0.064

CI: confifidence interval; APACHE II: acute physiology and chronic health evaluation II; SOFA: sequential organ failure assessment; CCI: the Charlson comorbidity index; eGFR: estimated glomerular filtration rate.

## Discussion

AKI is defined by a rapid increase in serum creatinine, decreased urine output, or both [20]. Since the KDIGO guideline for AKI was published in 2012 [11], substantial advances in our understanding of AKI epidemiology, pathophysiology, and diagnostic testing have fueled a growing controversy. However, the concept of AKI staging has clear and significant limitations that should be addressed, as it has relied on the established but poor biomarkers of solute clearance (serum creatinine levels and urinary output), and has been challenged by the identification of novel biomarkers of tubular stress and damage. However, the AKI criteria continue to be valuable, when no acceptable alternative was available [21].

The present study demonstrates that while stage 1b has the better basic renal function (higher eGFR), we found that the two subgroups differed significantly only in ICU LOS, however the two Scr_ref_ criteria (53 µmol/L or 61.88 µmol/L) in KDIGO AKI stage 1 did not distinguish the two associated populations in ICU mortality or RRT support. Furthermore, we could not establish an independent association of reclassification of stage 1 to ICU mortality. Respiratory failure and vasoactive drug therapy were found to be independently associated with the increased risk for death. Our results differed from that of a recent study [14], in which Sparrow and his colleagues screened 81,651 patients admitted to a large academic medical center and 4 satellite community hospitals. To operationalize the proposed 4-stage criteria correctly, they used linear regression and determined that the lower bound for Scr_ref_ was 61.88 µmol/L, and the LOS for stage 1b was longer than stage 1a. Moreover, in-hospital mortality was found to increase as the severity of AKI increased. Patients with AKI stages 1a and 1b experienced clinically significant differences in the LOS and mortality.

Our results showed no differences in mortality based on refined staging KDIGO_Scr_-AKI in ICU patients. There are several possible explanations for this. First, the Scr_ref_ exclusion criteria were different. In the study from Sparrow et al., patients’ data were used for linear regression to determine Scr_ref_, but in our study, Scr_ref_ was calculated according to the KDIGO definition. This value will remain invariable to changes in different research methods. In 2020, Lee et al. retrospectively analyzed AKI patients after liver transplantation [22] and planned to use Scr_ref_ 53 µmol/L as the lower bound to distinguish between stage 1a and 1b. However, due to the small number of patients with Scr_ref_ 53 µmol/L, 61.88 µmol/L was finally selected for the study. Interestingly, when Scr_ref_ of 61.88 µmol/L was selected, the patients with Scr_ref_ 53 to 61.88 µmol/L would be missed. In our study, it accounts for 22.61% of AKI patients and the statistical difference between Scr_peak_ 1b and Scr_peak_ 1a is significant. Second, the characteristics of ICU patients are different from those in the general ward. ICU patients may be admitted because of acute respiratory distress syndrome, septic shock, multiple trauma and many other different reasons, and there is often multiple organ damage. Although studies have found that a 1.5-fold increase in Scr during steady state conditions reflects a 39% decrease in eGFR [23], and the mortality rate increased to 6% in patients whose Scr levels increased to 44.2 µmol/L [24], but the pooled mortality rises to 42% in critically ill patients with KDIGO stage 3 and 46% of those requiring RRT [25]. KDIGO AKI stage 1, as the early impairment of a single organ can predict functional changes but which might not quantify damage, is unlikely to affect mortality or RRT ratio. Mortality in the Sparrow’s study was 49.7%, while in our study was 13.91%. This reduction in the total number of deaths in each stage, make it more difficult to find statistical differences in the current study.

There are some limitations to our study. First, this was a retrospective analysis of prospectively collected data, the database has been established for a long time, and the sample size was limited so we failed to analyze stage 3; unknown confounders could have affected the study results. The results of our study should be interpreted cautiously and require application in much larger ICU populations to elucidate further whether significant differences exist.

To conclude, we explored the characteristics and prognosis of KDIGO_SCr_ AKI stage 1. We found that stage 1a patients was beneficial in terms of ICU LOS compared to stage 1b when Scr_ref_ is 53 µmol/L or higher, but stage 1a patients had not decreased ICU mortality and RRT support.

## Data Availability

The datasets and resources analyzed during the current study are available from the corresponding author upon reasonable request.
